# Influence of the acid–base stoichiometry and residual water on the transport mechanism in a highly-Brønsted-acidic proton-conducting ionic liquid

**DOI:** 10.1039/d0ra08969a

**Published:** 2020-11-24

**Authors:** Jingjing Lin, Carsten Korte

**Affiliations:** Forschungszentrum Jülich GmbH, Institute of Energy and Climate Research - Fuel Cells (IEK-14) Wilhelm-Johnen-Straße 52425 Jülich Germany c.korte@fz-juelich.de; RWTH Aachen University 52062 Aachen Germany

## Abstract

In this study, Brønsted-acidic proton conducting ionic liquids are considered as potential new electrolytes for polymer membrane fuel cells with operating temperatures above 100 °C. *N*-Methyltaurine and trifluoromethanesulfonic acid (TfOH) were mixed at various stoichiometric ratios in order to investigate the influence of an acid or base excess. The proton conductivity and self-diffusion of the “neat” and with 6 wt% water samples were investigated by following electrochemical and NMR methods. The composition change in the complete species and the relative proton transport mechanism based on the NMR results are discussed in detail. During fuel cell operation, the presence of significant amounts of residual water is unavoidable. In PEFC electrolytes, the predominating proton transfer process depends on the cooperative mechanism, when PILs are fixed on the polymer matrix within the membrane. Due to the comparable acidity of the cation [2-Sema]^+^ and the hydroxonium cation, with excess *N*-methyltaurine or H_2_O in the compositions, fast proton exchange reactions between the protonated [2-Sema]^+^ cation, *N*-methyltaurine and H_2_O can be envisaged. Thus, an increasing ratio of cooperative proton transport could be observed. Therefore, for polymer membrane fuel cells operating at elevated temperatures, the highly acidic PILs with excess bases are promising candidates for future use as electrolytes.

## Abstract 2

There is a transition between prevailing vehicular and cooperative transport mechanism in base-excess Brønsted-acidic proton-conducting ionic liquids depending on stoichiometry.

## Graphical abstract

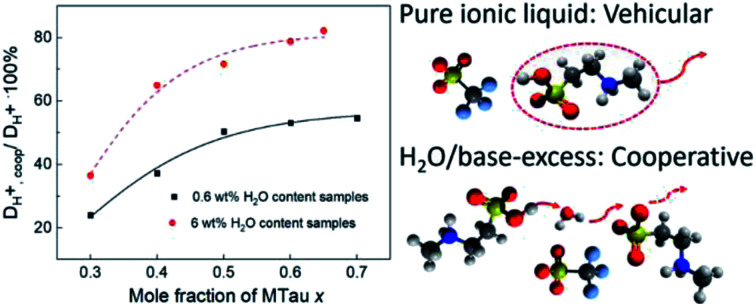

## Introduction

Polymer electrolyte fuel cells (PEFCs) operating at elevated temperatures (>100 °C) offer significant improvements over low-temperature PEFCs, such as no humidification of the feed gas, no water recirculation, a more efficient cooling of the cell and a higher tolerance against feed gas impurities.^1–3^ The proton conductivity of NAFION®-based proton exchange membranes (PEMs), used in PEFCs for low operation temperatures, depends mainly on the polymer's water uptake. For operation at elevated temperatures (>100 °C), the conductivity of a new membrane material should be maintained in anhydrous conditions. Currently, (high temperature-) HT-PEFCs are based on polybenzimidazole (PBI) membranes doped with phosphoric acid (H_3_PO_4_).^4–7^ However, the presence of H_3_PO_4_ leads to slow cathodic oxygen reduction reaction (ORR) kinetics.^8^ There is a specific adsorption of H_3_PO_4_ species on active sites in the redox catalyst platinum, which causes an inhibition (poisoning) effect.^9,10^ In addition, the insufficient solubility and diffusivity of oxygen is discussed.^11^ Thus, there is a need for new non-aqueous proton-conducting electrolytes to be operational for temperatures of 100–120 °C.

Proton-conducting ionic liquids (PILs) are promising candidates as non-aqueous electrolytes at operating temperatures >100 °C. Ionic liquids (ILs) are ionic compounds with bulky cations and anions and thus a low lattice energy.^12^ PILs have received much attention as a potential electrolyte in PEFCs due to their good conductivity, wide electrochemical windows and low flammability.^13–18^ In a PIL, the cation or anion may act as a protonic charge carrier, and so either the cations or anions are Brønsted-acids. In the case of cations, *i.e.*, a PIL of the type HB^+^A^−^, it consists of an (organic) base B, protonated by a very strong acid HA, respectively a super acid:

1 B + HA ⇄ HB^+^ + A^−^

The anions of super acids, such as trifluoromethanesulfonic acid or bis-trifluoromethylsulfonimid, have a less inhibiting effect on electrocatalytically-active electrode surfaces than H_3_PO_4_. The triflimid (CF_3_SO_2_)_2_N^−^ and triflate CF_3_SO_3_^−^ anions interact only very weakly with metal atoms, resulting in weak adsorption on a Pt surface.^9,19,20^ In a water-free PIL of the type HB^+^A^−^, protons can only move in an electric field *via* the protonated cations HB^+^ by means of a vehicle mechanism. A drawback of ILs or PILs is often poor conductivity because of relatively high viscosity. A proton transfer back to the anion of the superacid, *e.g.*, [TfO]^−^, has only a very small probability because the protolysis equilibrium in eqn (1) is on the far right side. Thus, a cooperative transport mechanism involving the anions is not possible. In the case of vehicular mechanism, conductivity and viscosity are coupled to each other according to the Stokes–Einstein relation.

However, to avoid the leakage of the liquid electrolyte during operation, a PIL applied in a PEFC must be immobilized in a polymer matrix. A study of a [Dema][TfO]-doped PBI membrane by Liu *et al.* shows an activation energy of the conductivity in the range of the cooperative mechanism,^21^ whereas the pure [Dema][TfO] is vehicular.^22^ In this case, the vehicular transport of the cation HB^+^ is constrained and a cooperative proton transport mechanism would be advantageous. This was shown in a study by Noda *et al.* that the excess in the base B of a PIL of the type HB^+^A^−^, which provides the cooperative proton transport and improves conductivity.^12^ The excess base B acts as a proton acceptor that is protonated by the proton donor HB^+^. The cooperative transports through the excess base B only necessitate the reorientations of the involved particles B an HB^+^. This results in increased proton conductivity and reduced activation energy for the conduction process.^12,23,24^ In particular, PILs contain strong Brønsted-acidic cations that are usually highly hygroscopic. The water absorption is difficult to prevent. Moreover, under fuel cell operation, water will be generated on the cathode side. The presence of residual water acts as a proton acceptor and gives rise to a protolysis equilibrium with the cation HB^+^:

2 HB^+^ + H_2_O ⇄ B + H_3_O^+^

Its extent depends on the acidity of the cation. In a preceding NMR study, it was shown that in Brønsted-acidic PILs of the type HB^+^A^−^ cooperative proton transfer will dominate, depending on the cation acidity and the residual water content.^25^ There will also be fast exchange between HB^+^, B, H_2_O and H_3_O^+^. In general, as discussed above, the introduction of a proton acceptor would improve the cooperative transport. The coexistence of excess base B and residual water offer the possibility to improve the technically utilizable conductivity of PIL electrolytes.

In this experimental study, the effect of the PIL acid–base stoichiometry on the proton transport mechanism in a system with residual water is investigated. In general, cooperative proton transport in an IL system requires the presence of a proton acceptor and proton donor with comparable acidity.^26^ A highly acidic PIL, 2-sulfoethylmethylammonum triflate [2-Sema][TfO] (p*K*_A1_ ≈ −1),^27^ is used. The acidity of this is comparable to the hydroxonium cation (p*K*_A_ = 0). The 2-sulfoethylmethylammonium cation is prepared by means of the protonation of 2-methylaminoethanesulfonic acid (*N*-methyltaurine), which exists as a zwitterion due to tautomerism. Because of the presence of the sulfonic acid functionality, it is a very strong acid that can protonate the residual water at a significant percentage.^8^

Appropriate amounts of *N*-methyltaurine (MTau) and trifluoromethanesulfonic acid (TfOH) are mixed at various molar ratios to vary the PILs compositions from the TfOH-excess to MTau-excess. The interactions between the cations, the excess base and H_2_O are determined by means of electrical conductivity measurements and ^1^H NMR spectroscopy. Using a pulsed-field gradient (PFG-) NMR technique, the self-diffusion coefficients of the individual protons in the PILs are obtained. The effect of stoichiometry and residual H_2_O on the prevailing proton transport mechanism^26^ is discussed by comparing the measured macroscopic and microscopic properties.

## Experimental

### Materials

Equimolar 2-sulfoethylmethylammonum triflate [2-Sema][TfO] is prepared by slowly adding trifluoromethanesulfonic acid (triflic acid, reagent grade: 98%, Sigma Aldrich) to 2-methylaminoethanesulfonic acid (*N*-methyltaurine, ≥99%, Sigma Life Science). In the following, *N*-methyltaurine is abbreviated as MTau in analogy to triflic acid (TfOH). The total amount of [2-Sema][TfO] is divided into 5 bottles, each of about 5 g. Appropriate amounts of TfOH and *N*-methyltaurine are added to each bottle to maintain the defined molar ratios *x*, *i.e.*, *x*[MTau]·(1 − *x*)[TfOH]. The mole fraction of *N*-methyltaurine was from *x* = 0.3–0.7. Thus, after mixing and heating samples with the composition *x*[2-Sema][TfO]·(1 − 2*x*)[TfOH] for *x* = 0.3–0.5 and with the composition (1 − *x*)[2-Sema][TfO]·(2*x* − 1)[MTau] for *x* = 0.5–0.7 are obtained.

**Fig. 1:**
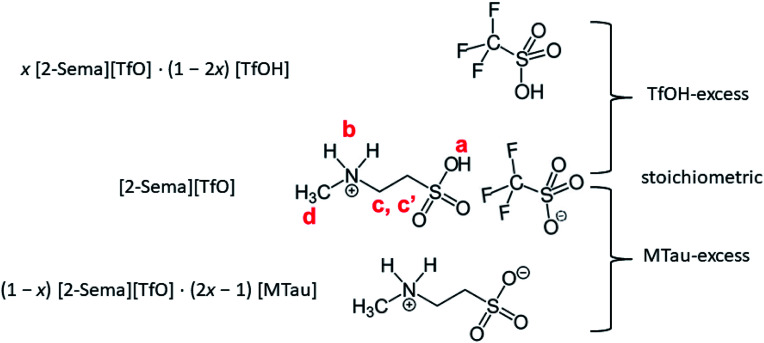
*x*[MTau]·(1 − *x*)[TfOH] samples with an excess of the acid TfOH or the base *N*-methyltaurine.

Using Karl-Fischer titration, it can be observed that the samples prepared from the starting materials have a water content of 0.75–0.8 wt%. To analyze the water content, two series of samples were investigated. The first series were the as-prepared (neat) *x*[MTau]·(1 − *x*)[TfOH] samples with a residual water content of 0.75–0.8 wt% and the second series were samples diluted with water to an H_2_O content of 6 wt%, *i.e.*, *x*[MTau]·(1 − *x*)[TfOH] + 6 wt% H_2_O. The (molar) ratio of *x*[MTau]·(1 − *x*)[TfOH] : H_2_O is in the range of 0.98–1.0, due to the similar molar mass between *M*_Mtau_ = 139 and *M*_TfOH_ = 150. The different stoichiometries of the samples are illustrated in Fig. 1. The samples with the composition *x* = 0.3–0.5 have a “TfOH-excess”. Conversely, the samples with the composition *x* = 0.5–0.7 (or 0.65) have an “MTau-excess”.

### Conductivity measurement

The AC conductivity measurements were performed in a four-probe conductivity cell, using platinum electrodes. The cell constant as a function of the sample volume was determined by using a 0.1 M KCl solution for calibration. The intended water contents of the binary PIL + H_2_O mixtures were checked using Karl-Fischer titration at the beginning of each measurement. The total ohmic resistance *σ* as a function of the temperature *T* of the neat PIL and of the PIL + H_2_O samples was determined by means of impedance spectroscopy. The temperature *T* was increased in increments of 10 °C from 60 to 110 °C and *vice versa*. The excitation amplitude was adjusted to 10 mV. The specific conductivity *σ* was calculated by using the cell constant.

### ^1^H NMR parameters

The acquisition of the NMR spectra was performed using a Bruker 600 MHz spectrometer, equipped with a 5 mm cryoprobe tuned to ^1^H. A capillary filled with D_2_O was enclosed the sample tubes as a field lock. The measurements were performed at 90 °C, because at lower temperatures, the increasing viscosity leads to high relaxation times.

### Measurement of the self-diffusion coefficients

The self-diffusion coefficients of the observable protons were measured using the diffusion-ordered spectroscopy (DOSY) technique at 90 °C. The measurements were performed by applying 30 field gradient increments with a gradient strength *g* from 1.3 to 32.5 G cm^−1^. The values of the gradient pulse length *δ* and the diffusion time intervals *Δ* were optimized to aim for least 85% signal attenuation at the strongest field gradient. The value of the (self-) diffusion coefficient *D*_i_ of a certain proton species i was obtained from the decay of its measured echo intensity *vs.* the gradient field strength *g*.

## Results and discussion

### Total conductivity *vs.* stoichiometry and water content

The measurements of the total conductivity *σ* were performed in the temperature range between 60 and 110 °C. The total conductivity includes cationic and anionic charge transport. In the *x*[MTau]·(1 − *x*)[TfOH] and the *x*[MTau]·+(1 − *x*)[TfOH] + 6 wt% H_2_O samples, the viscosity was strongly dependent on the composition. In general, the viscosity was rising with an increasing fraction *x* of the (at room temperature solid) base *N*-methyltaurine, *i.e.*, with increasing MTau-excess. In the case of an increasing TfOH-excess, the viscosity was generally decreasing, *i.e.*, with decreasing *x*. A higher content of H_2_O also leads to a lower viscosity. The dependence of the total conductivity *σ*, respectively of the specific total conductivity *Λ* on the viscosity *η*, can be explained according to the Stokes–Einstein and Nernst–Planck relations:3
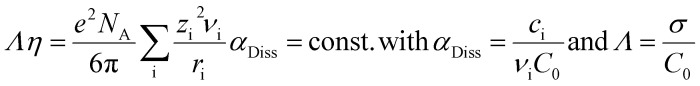


Assuming an ionic compound A_*ν*_1__B_*ν*_2__C_*ν*_2__…X_*ν*_i__, *ν*_i_ denotes the stoichiometric factor, *z*_i_ the charge number and *r*_i_ the hydrodynamic radius of the ionic species i. In the case of a dissociation degree *α*_Diss_ not being equal to unity, the concentration of the ionic species i is denoted with *c*_i_ and the initial concentration of the ionic compound A_*ν*_1__B_*ν*_2__C_*ν*_2__…X_*ν*_i__ with *c*_0_. Thus, a decrease in the viscosity will accelerate the vehicular proton transport by PIL cations and H_3_O^+^.

**Fig. 2:**
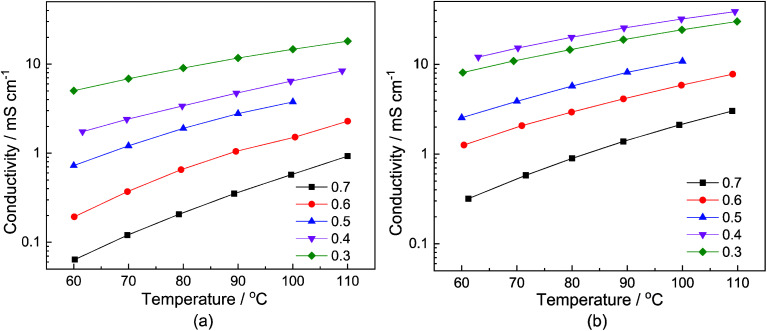
The conductivity of: (a) neat *x*[MTau]·(1 − *x*)[TfOH] (0.75–0.8 wt%); and (b) *x*[MTau]·(1 − *x*)[TfOH] + 6 wt% H_2_O samples as a function of the temperature. The molar fraction of *N*-methyltaurine varies from *x* = 0.3 to 0.7.

The dependency of the total conductivity *σ* on the neat samples and samples with a water content of 6 wt% on the temperature *T* and the stoichiometry *x* is depicted in Fig. 2(a) and (b), respectively. The course of the conductivities corresponds to the change in the viscosity. In the case of the neat samples, depicted in Fig. 2(a), the sample with the smallest MTau molar fraction *x* = 0.3 exhibits the highest total conductivity. Correspondingly, the total conductivities of the samples with a water content of 6 wt% are generally higher compared to the neat sample, as is depicted in Fig. 2(b).

For all of the investigated samples, the total conductivity *σ* increases as a function of the temperature *T*. However, the extent of the conductivity increase *vs.* temperature is different. The highest impact on the conductivity was found for the sample with the highest MTau molar fraction of *x* = 0.7. For this MTau molar fraction, in the case of the neat samples, *σ* increases by a factor of 8.4 when *T* rises from 60 °C to 110 °C. In the case of the sample with a molar fraction of *x* = 0.3, the increase was only by a factor of 3. The samples with a water content of 6 wt% and a molar fraction of *x* = 0.7 and *x* = 0.3 show a similar behaviour, with the conductivity increasing by a factor of 8.52 and 2.75, respectively. The different factors may indicate that there is not only a change in viscosity but also of the proton transport mechanism responsible for the increase in the total conductivity when increasing the molar fraction of MTau. The total conductivity describes the bulk charge transport macroscopically and includes all mobile charge carriers ([2-Sema]^+^, [TfO]^−^ and H_3_O^+^). The underlying ionic charge (proton) transport mechanisms can only be discerned by techniques sensitive to the local environment of the mobile charge carriers, *i.e.*, the NMR.

### ^1^H-NMR of neat *x*[MTau]·(1 − *x*)[TfOH] samples

In the following, ^1^H-NMR and ^1^H-PFG-NMR are used to measure the local dynamics of the mobile protonic charge carriers, as well as their self-diffusion coefficients. As discussed above, in a PIL of the type HB^+^A^−^, an excess of the base B as well as a certain water content are able to provide cooperative transport and thus improve the proton conductivity. Therefore, depending on the excess of the base and water content, both transport mechanisms, vehicular and cooperative, may be present in a sample.

**Fig. 3:**
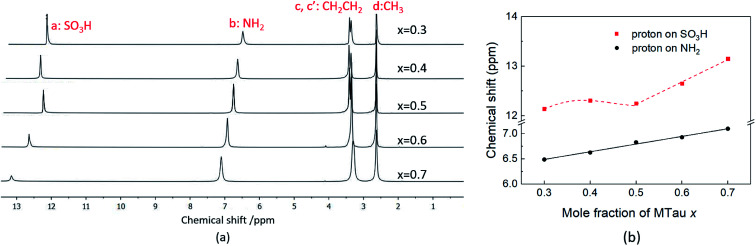
(a) The ^1^H NMR spectra and (b) the ^1^H NMR spectra chemical shift (ppm) of SO_3_H and NH_2_ of neat *x*[MTau]·(1 − *x*)[TfOH] at 90 °C.

Due to the high viscosity of the samples at room temperature, especially in the case of MTau-excess, the NMR measurements were all performed at 90 °C to avoid an FWHM of the signals too broad to evaluate. The ^1^H NMR spectra of *x*[MTau]·(1 − *x*)[TfOH] (*x* = 0.3–0.7) at a temperature of 90 °C are depicted in Fig. 3(a) and the chemical shift *δ* of the protons situated in the SO_3_H and NH_2_^+^ group *vs.* the stoichiometry *x* in Fig. 3(b). The signals of the protons are labelled with (a), (b), (c, c′) and (d), according to Fig. 1. In the case of the stoichiometric [2-Sema][TfO] sample, *i.e.*, *x* = 0.5, the protons of the NH_2_^+^ group (b) show up at a chemical shift *δ* of 6.83 ppm and the proton of the SO_3_H group (a) at 12.25 ppm. When increasing the mole fraction *x* of the base MTau from 0.3 to 0.7, the signal (b) of the NH_2_^+^ protons shifts about 0.6 ppm towards the lower magnetic field, *i.e.*, from a chemical shift of 6.49 to 7.10 ppm. In the case of SO_3_H protons (a) there is a slight shift of +0.06 ppm towards lower magnetic fields when increasing *x* from 0.3 to 0.4 and a slight shift of −0.17 ppm towards higher magnetic fields from 0.4 to 0.5. Beyond the stoichiometric composition, for a molar fraction *x* of 0.5 to 0.7, the signal shifts about +0.9 ppm towards lower magnetic fields with increasing the base excess. The signal of the protons of the CH_3_ group (d) show up at a chemical shift *δ* of 2.63 ppm and of the CH_2_CH_2_ backbone (c, c′) at about 3.37 ppm. There is no appreciable shift in the CH_3_ (d) and the CH_2_CH_2_ (c, c′) protons. In the case of samples with a base-excess, *i.e.*, *x* > 0.5, the protons of the CH_2_CH_2_ backbone (c, c′) are difficult to distinguish due to a general increase of the FWHM in the spectra.

**Fig. 4:**
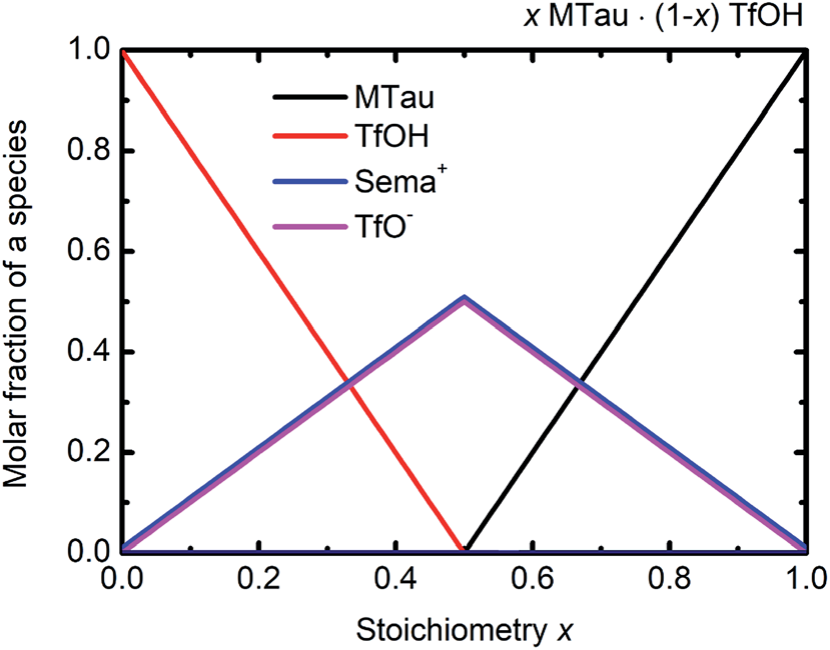
Molar fractions of the species [2-Sema]^+^, MTau, TfOH and [TfO]^−^ as a function of the stoichiometry *x* for the samples *x*MTau·(1 − *x*)TfOH. The curves of [2-Sema]^+^ and [TfO]^−^ coincide.

The NMR chemical shift *δ* of a proton depends on the local screening of the external magnetic field by the local electron density. In the timescale of the NMR experiment, a chemical shift to a lower field represents a generally lower local electron density or higher “delocalization” of the proton. The local electron density can be affected by intramolecular interactions with adjacent groups or intermolecular interactions by hydrogen bonds. A high acidic proton is accompanied by a low local electron density. In addition, the formation of a hydrogen bond leads to the deshielding of a proton. Thus, the (NMR-) active protons of a Brønsted-acidic PIL in particular are influenced not only by the water content but also by the acid–base stoichiometry, as the active protons can easily form hydrogen bonds and are subject to intramolecular interactions. In the case of the water-free samples, the molar fractions of the species [2-Sema]^+^, MTau, TfOH and [TfO]^−^ should vary as a function of the stoichiometry *x*, as depicted in Fig. 4. TfOH is a much stronger acid compared to the [2-Sema]^+^ cation, and so we can safely assume a complete proton transfer from TfOH to MTau for the entire range of stoichiometry *x*. This should lead to a decreasing fraction of TfOH in the stoichiometric range from *x* = 0 to 1/2 and an increasing fraction of MTau in the range from *x* = 1/2 to 1.

As the acidity of the NH_2_^+^ moiety in the [2-Sema]^+^ cation and MTau is not sufficient to protonate SO_3_^−^ moieties (intra/intermolecular) to a noticeable extent, the residence time prior to re-transfer should be very short. Thus, protolysis equilibria should not contribute to the observed stoichiometry *x*-dependent shift of these protons (p*K*_A2_ of NH_2_^+^ in MTau, about 10.2).^27–29^ As is shown in Fig. 4, the sum of the molar fractions of [MTau] and [TfO]^−^ is increasing when increasing *x* from 0.3 to 0.7. Thus, in the case of the NH_2_^+^ protons, only the probability of forming H-bonds with neighboring [TfO]^−^ anions and [2-Sema]^+^ cations and excess MTau molecules, respectively, can be considered to explain the observation that the *δ* value of the NH_2_^+^ protons shifts monotonically towards lower fields with a slope of 0.15 ppm/0.1 Δ*x* (deshielding).

In the case of SO_3_H protons, a stronger effect can be observed compared to the NH_2_^+^ protons. Despite the fact that SO_3_H protons are present on [2-Sema]^+^ cations and TfOH, there is only one signal shifting from 12.14 to 13.15 ppm to lower fields. The sites cannot be distinguished on the NMR spectrum. In addition to the possibility of forming H-bonds, the possibility/frequency of intermolecular proton transfers must also be considered. In the case of MTau-excess, *i.e.*, for samples with a stoichiometry of *x* = 0.5 to 0.7, there will be only variable fractions of [2-Sema]^+^, MTau and [TfO]^−^. The acidity of TfOH (p*K*_A_ = −14) is much higher than that of [2-Sema]^+^ (p*K*_A_ = −1).^27^ The proton transfer from the cation [2-Sema]^+^ back to the [TfO]^−^ anion will not take place to a noticeable degree. Thus, TfOH is fully deprotonated. There are only fast intermolecular proton transfers between SO_3_^−^ moieties of [2-Sema]^+^ and MTau, resulting in the strong deshielding of the proton with increasing *x*:

4 CH_3_NH_2_^+^(CH_2_)_2_SO_3_**H** + CH_3_NH_2_^+^(CH_2_)_2_SO_3_^−^ → CH_3_NH_2_^+^(CH_2_)_2_SO_3_^−^ + CH_3_NH_2_^+^(CH_2_)_2_SO_3_**H**

In the stoichiometry range between *x* = 0.5 to 0.7, the signal shifts with a slope of 0.45 ppm/0.1 Δ*x*; see Fig. 4.

In the case of TfOH-excess, *i.e.*, for samples with a stoichiometry of *x* = 0.3 to 0.5, the shift in the signal is comparably small and not monotonic. Due to the TfOH excess, the SO_3_H moiety of the MTau is fully protonated. There are only variable fractions of [2-Sema]^+^ cations, TfOH and [TfO]^−^. Thus, there is primarily an intermolecular proton transfer between the anion [TfO]^−^ and TfOH, which also results in a de-shielding of the proton:

5 CF_3_SO_3_**H** + CF_3_SO_3_^−^ → CF_3_SO_3_^−^ + CF_3_SO_3_**H**

Changing the stoichiometry from *x* = 0.5 to 0.4 leads to an increasing delocalization of the SO_3_H proton and to an initial downfield shift, as seen in the NMR chemical shift in Fig. 3(b). In the system TfOH/[TfO]^−^, the presence of [TfO]^−^ should be equivalent to the excess base MTau in the system [2-Sema]^+^/MTau. Thus, a further increase in the molar fraction of TfOH may inhibit the delocalization again, resulting in the observed shift of the SO_3_H proton back towards the higher field when changing the stoichiometry from *x* = 0.4 to 0.3, due to the restricted mobility.

In the case of the neat, nearly water-free samples, a change in the stoichiometry will affect the probability and duration of forming hydrogen bonds to the NH_2_ proton on cations in the timescale of the NMR measurement. In the case of the SO_3_H proton, the MTau-excess leads to a significant delocalization between the SO_3_^−^/SO_3_H sites in the timescale of the NMR measurement and TfOH-excess results to a delocalization between the [TfO]^−^/TfOH. Both of these may explain the observed signal shift to high magnetic fields in the ^1^H NMR spectrum. The acid–base stoichiometry also influences the proton transport processes. The observation of a proton delocalisation on the NMR timescale indicates the possible presence of intermolecular (cooperative) proton transport. The influence of the proton delocalization on the proton transport will be discussed in relation to the self-diffusion coefficient in the section “^1^H-PFG-NMR”.

### ^1^H-NMR of *x*[MTau]·(1 − *x*)[TfOH] samples with 6 wt% water

**Fig. 5:**
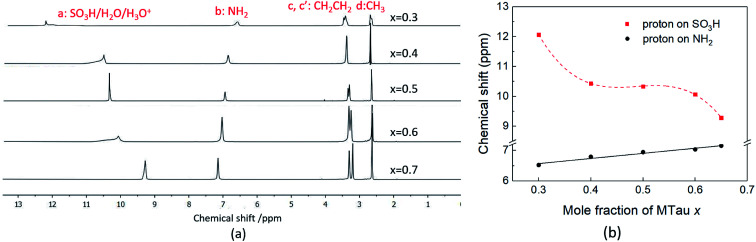
(a) The ^1^H NMR spectra; (b) the ^1^H NMR spectra chemical shift (ppm) of SO_3_H and NH_2_ of 6 wt% water concentration *x*[MTau]·(1 − *x*)[TfOH] + 6 wt% H_2_O at 90 °C.

The ^1^H NMR spectra of *x*[MTau]·(1 − *x*)[TfOH] (*x* = 0.3–0.65) with a water content of 6 wt% at 90 °C is depicted in Fig. 5(a) and the chemical shift *δ vs.* stoichiometry *x* of the of SO_3_H and NH_2_^+^ protons in Fig. 5(b). Similar to the ^1^H NMR spectrum of (neat) *x*[MTau]·(1 − *x*)[TfOH] samples, the protons of the CH_3_ group in *x*[MTau]·(1 − *x*)[TfOH] + 6 wt% H_2_O can be found at 2.63 ppm. The protons of the CH_2_CH_2_ backbone (c, c′) appear as two separated signals in most of the samples. These can be found at about 3.30 and 3.25 ppm. When increasing the MTau mole fraction, the signal of the NH_2_^+^ protons also shifts towards a lower magnetic field, as is observed for the neat samples. It shifts from 6.52 to 7.15 ppm (about +0.6 ppm) when changing *x* from 0.3 to 0.65. A higher water concentration will generally increase the number of species available to form H-bonds (leading to a deshielding), which may provide an additional shift of ∼+0.05 ppm for all stoichiometric compositions compared to the (neat) *x*[MTau]·(1 − *x*)[TfOH] samples. The slope of 0.18 ppm/0.1 Δ*x* is slightly higher.

In the investigated stoichiometry range from *x* = 0.3 to 0.65, the signal of the SO_3_H proton shifts in the opposite direction compared to the (neat) *x*[MTau]·(1 − *x*)[TfOH] samples, *i.e.*, towards higher fields (from 12.1 to 9.3 ppm). The additional H_2_O in *x*[MTau]·(1 − *x*)[TfOH] + 6 wt% H_2_O samples acts as another proton acceptor. Depending on the stoichiometric composition *x*, it can be protonated by the very high acidic excess TfOH or by the high acidic [2-Sema]^+^ cation according to the protolysis equilibria shown in eqn (6) and (7):6
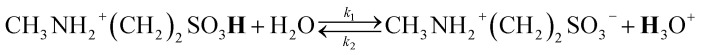

7 TfO**H** + H_2_O → TfO^−^ + **H**_3_O^+^

TfOH is the much stronger acid compared to the [2-Sema]^+^ cation and the acidity of the hydroxonium cation H_3_O^+^ is on the same order as the acidity of the [2-Sema]^+^ cation.††The p*K*_A_ of H_3_O^+^ is equal to 0, the p*K*_A_ of typical alkane sulfonic acids is about −1.^30,31^ A preceding work confirms the fast exchange of the H_2_O proton and SO_3_H proton, resulting in a single signal in the spectrum of SO_3_H/H_3_O^+^/H_2_O at an average NMR shift.^25^ Moreover, the signal of the SO_3_H proton shows a corresponding increase in its integral area with increasing H_2_O content, which is not the case for the NH_2_^+^ or alkyl protons.

**Fig. 6:**
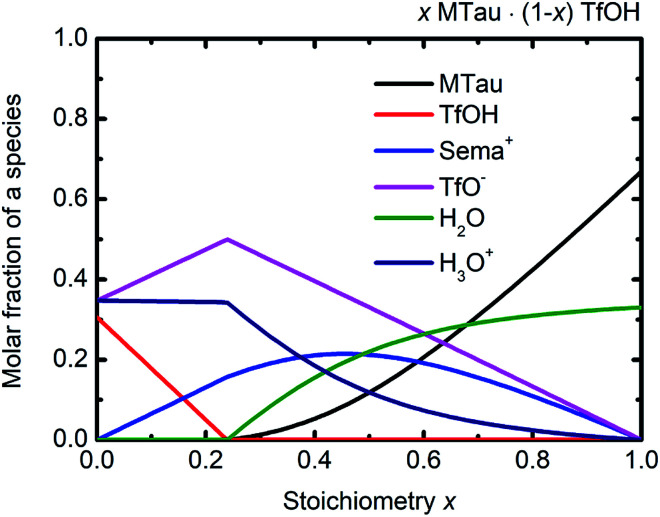
(Tentative) molar fractions of the species [2-Sema]^+^, MTau, TfOH, [TfO]^−^, H_3_O^+^ and H_2_O as a function of the stoichiometry *x* for the samples *x*MTau·(1 − *x*)TfOH + H_2_O.

Considering the protolysis reactions in eqn (6) and (7), below a stoichiometry *x* of 0.24, all existing MTau and H_2_O will be protonated by the excess TfOH in the *x*[MTau]·(1 − *x*)[TfOH] + 6 wt% H_2_O samples.‡‡This corresponds to an initial molar fraction of 0.50 for TfOH, 0.16 for MTau and 0.34 for H_2_O, considering the molar weights and a water content of 6 wt%. Part of the excess TfOH should be undissociated. There will only be [2-Sema]^+^, H_3_O^+^, TfOH and [TfO]^−^ in the samples. At a stoichiometry *x* above 0.24, unprotonated MTau and H_2_O should be present, as all TfOH is dissociated, *i.e.*, only [TfO]^−^ should exist in this range. With an increasing fraction of H_2_O, a portion of the [2-Sema]^+^ cations can also be deprotonated. Taking the preceding investigations of the analog compound 2-sulfoethylammonium triflate into account, a protolysis degree of the [2-Sema]^+^ cation of about 0.3 can be estimated for a sample with a stoichiometric composition (*x* = 0.5).^32^ These assumptions are underpin the construction of the tentative plot in Fig. 6.

In the case of the investigated *x*[MTau]·(1 − *x*)[TfOH] + 6 wt% H_2_O samples with a stoichiometry of *x* = 0.3 to 0.65, the very high acidic TfOH should always be fully consumed by protonating the H_2_O molecules and forming H_3_O^+^ cations, respectively, by protonating MTau, forming [2-Sema]^+^ cations; see Fig. 6. Thus, there will only be [2-Sema]^+^, MTau, H_3_O^+^, H_2_O and [TfO]^−^. Here, the intermolecular transfer between TfOH and [TfO]^−^, as stated in eqn (5), would not take place. As MTau will act as a proton acceptor, the equilibrium fractions of H_2_O, H_3_O^+^ and [Sema^+^] cations depend on the stoichiometry *x* according eqn (6) and (7). Increasing the stoichiometry *x* from 0.3 to 0.65 should lead to a decreasing fraction of H_3_O^+^ and an increasing fraction of H_2_O. The fraction of the [2-Sema]^+^ cations will reach a maximum in close proximity to the stoichiometric point *x* = 1/2. There is a delocalization of the active proton in the NMR timescale between H_2_O, H_3_O^+^, MTau and [2-Sema]^+^, because the neutral MTau is competing with H_2_O for the protons of the [2-Sema]^+^ and H_3_O^+^ cations. The chemical shift found for the H_3_O^+^ protons (∼9.6–10.9 ppm) is at a much lower magnetic field compared to H_2_O (∼3–5 ppm).^33^ Thus, the averaged ppm value of the SO_3_H/H_3_O^+^/H_2_O proton should also decrease, respectively shifting towards a higher field, as it inclines to the ppm value of H_2_O; see Fig. 5.

The proton transport mechanism is further discussed, together with the ^1^H-PFG-NMR/DOSY measurements and the self-diffusion coefficient in the next section. The mobile protonic charge carriers in the PIL/H_2_O system are [2-Sema]^+^ and, due to protolysis, also the H_3_O^+^ cations. In the case of vehicular transport, the H_3_O^+^ cations are probably much more mobile than the [2-Sema]^+^ ones. For a stoichiometry *x* > 0.3, the fractions of H_3_O^+^ and [TfO]^−^ monotonously decrease with increasing *x*. The fraction of [2-Sema]^+^ reaches a maximum at *x* ≈ ½. If there is only vehicular transport, this should principally lead to a decrease in the (total) conductivity with increasing *x*. If there is also cooperative transport, the presence of H_2_O, acting as a proton acceptor, will also accelerate the intermolecular proton transfer between the MTau and the [2-Sema]^+^ cation, leading to faster cooperative transport. For a stoichiometry of *x* > 0.3, the fraction of (free) H_2_O increases with increasing *x*. A maximum of an additional cooperative transport and thus of the (total) conductivity should be expected when the equimolar fractions of H_2_O and H_3_O^+^ are present (the maximum probability for proton transfers between H_3_O^+^ and H_2_O). This is approximately the case for a stoichiometry *x* ≈ 0.4 and corresponds well to the measured values for the total conductivity which exhibits a maximum at this stoichiometry; see Fig. 2(b) and 6. However, the total conductivity is highly coupled with the viscosity. At a higher stoichiometric composition *x* > 0.4, the vehicular transport is again attenuated and thus the conductivity is decreased.

### ^1^H-PFG-NMR/DOSY

The self-diffusion coefficient of the protons is measured by ^1^H-PFG-NMR at 90 °C. The diffusion coefficient *D*_H^+^_ of the (active) SO_3_H/H_2_O/H_3_O^+^ proton and *D*_cation_ of the other protons of the [2-Sema]^+^ cation/MTau are depicted in Table 1 for neat *x*[MTau]·(1 − *x*)[TfOH] and for *x*[MTau]·(1 − *x*)[TfOH] + H_2_O, *i.e.*, with a water content of 6 wt%. The value of the diffusion coefficient for the NH_2_ protons is nearly the same as for the CH_2_CH_2_ and CH_3_ protons. Thus, these can be identified as the diffusion coefficient *D*_cation_ of the entire cation, respectively, to the diffusion coefficient *D*_H^+^,vehicle_ of protons only by means of a vehicular mechanism. The diffusion coefficient *D*_H^+^_ of the SO_3_H/H_2_O/H_3_O^+^ protons is significantly higher compared to the diffusion coefficient *D*_cation_ of the [2-Sema]^+^ cation in all of the samples. As discussed, the observed intermolecular protons transfer between the SO_3_H moieties, H_2_O and H_3_O^+^, indicating the presence of cooperative transport and resulting in higher values of *D*_H^+^_ compared to *D*_cation_.

**Table 1:** Self-diffusion coefficients of the SO_3_H/H_2_O/H_3_O^+^ (≡*D*_H^+^_) and the cation protons in neat *x*[MTau]·(1 − *x*)[TfOH] and 6 wt% water concentration *x*[MTau]·(1 − *x*)[TfOH] + H_2_O at 90 °C

MTau molar fraction *x*	*D*_i_/10^−6^ cm^2^ s^−1^ in neat samples		*D*_i_/10^−6^ cm^2^ s^−1^ in samples with 6 wt% H_2_O	
	H^+^	Cation	H^+^	Cation
0.3	15.4 ± 0.7	11.7 ± 0.7	12.2 ± 1.3	7.20 ± 0.9
0.4	4.63 ± 0.4	3.00 ± 0.3	9.35 ± 1.3	3.15 ± 0.6
0.5	1.31 ± 0.0	0.66 ± 0.0	6.00 ± 0.1	1.65 ± 0.1
0.6	1.13 ± 0.2	0.56 ± 0.2	4.95 ± 0.4	1.05 ± 0.3
0.65	—	—	8.14 ± 0.4	1.51 ± 0.4
0.7	0.75 ± 0.1	0.34 ± 0.2	—	—

As observed for the total conductivity *σ*, the diffusion coefficient *D*_H^+^_ of the active proton and *D*_cation_ of the [2-Sema]^+^ cation are decreasing with increasing stoichiometry *x* (from 0.3 to 0.65, respectively, to 0.7). The dynamic viscosity *η* of the samples is directly coupled to the diffusion coefficient of the [2-Sema]^+^ cation *D*_cation_ due to the Stokes–Einstein relation. In the case of cooperative transport, there is a distinct decoupling from viscous processes. Thus, if vehicular and cooperative mechanisms are both present, an increase in viscosity will increase the impact of cooperative transport to the total proton transport. The share of cooperative processes in all of the samples is evaluated by calculating the ratio between *D*_H^+^,coop_ to *D*_H^+^_ as follows:^25^8
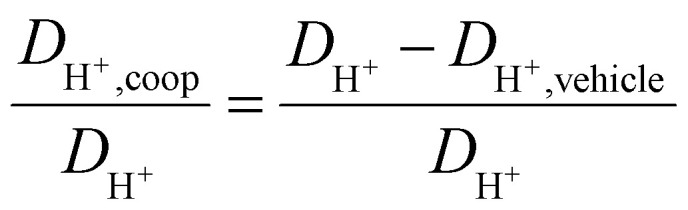


**Fig. 7:**
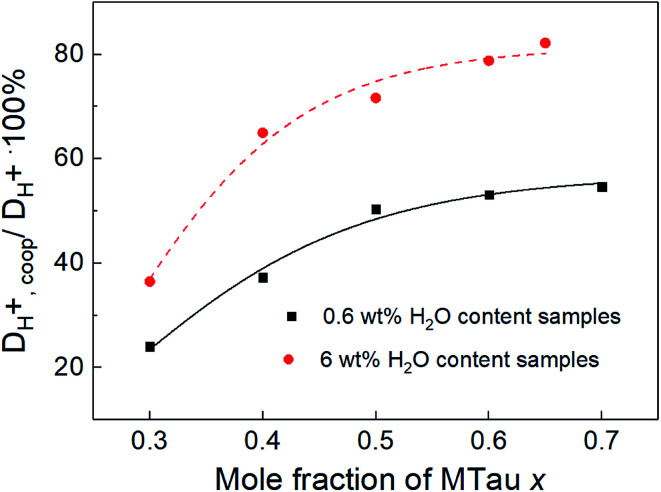
The ratio of *D*_H^+^,coop_ to *D*_H^+^_ in all of the measured samples.

The diffusion coefficient *D*_H^+^,vehicle_ for vehicular transport is identical to that of the [2-Sema]^+^ cation. The ratio represents the share of cooperative transport in the total proton diffusion process. The share of cooperative transport is depicted in Fig. 7. With increasing stoichiometry *x*, *i.e.*, with an increasing fraction of MTau, the share of cooperative transport is also increasing for the neat samples and for samples with a water content of 6 wt%. When comparing the course of both curves, as expected, the presence of the amphoter water generally increases the share of the cooperative mechanism. The ratio of *D*_H^+^,coop_ to *D*_H^+^_ reaches a value of 82% in a sample with a stoichiometry of *x* = 0.65 and 6 wt% water content. Corresponding to the chemical shift in the ^1^H-NMR spectrum, the ratio relates to the ability of the protons to be delocalized on the NMR timescale and thus to participate in intermolecular transfer. A higher share of cooperative transport generally leads to a lower field of the chemical shift, as discussed above. In the case of the high acidic PIL [2-Sema][TfO], due to the similar acidity of [2-Sema]^+^ and H_3_O^+^, the neutral MTau and H_2_O can both act as proton acceptors. The excess proton acceptors provide more sites for proton hopping, which accelerate the cooperative proton transport mechanism.

## Conclusion

*N*-Methyltaurine and TfOH were mixed at various molar ratios in order to obtain samples of the proton-conducting liquid [2-Sema][TfO] with various amounts of excess free acid TfOH or excess free base *N*-methyltaurine. The “nearly neat” samples and the samples with 6 wt% residual water were investigated regarding proton conductivity and self-diffusion using electrochemical and NMR methods. It could be observed that an excess of the free base MTau retards the vehicular proton transport due to an increase in the dynamic viscosity, which leads macroscopically to a lower (total) conductivity. Due to a comparable acidity of the cation [2-Sema]^+^ and the hydroxonium cation H_3_O^+^, an excess of MTau or H_2_O will enhance the fast proton exchange reactions between the [2-Sema]^+^ cation, H_3_O^+^, MTau and H_2_O and thus will increase the share of cooperative transport.

In an PEFC electrolyte, based on a PIL immobilised in a polymer matrix, the proton conductivity depends on the presence and the share of cooperative transport as vehicular transport is significantly hampered. The use of a base-excess high acidic PIL would allow a higher fraction of cooperative transport and thus a higher proton conductivity. For the future use as conductive electrolytes in PEFCs at elevated operation temperatures (100–120 °C) and atmospheric (non-humidified) operation, a PIL with a high hygroscopicity to retain H_2_O formed during operation at the cathode and an excess of the base may be favorable. Enabling fast cooperative transport may help in reaching sufficient proton conductivities.

## Conflicts of interest

There are no conflicts to declare.

## Supplementary Material
